# Obstructed hemivagina and ipsilateral renal agenesis: magnetic resonance imaging findings in young Nepali females – a report of four cases

**DOI:** 10.1097/MS9.0000000000000904

**Published:** 2023-05-24

**Authors:** Sharma Paudel, Shailendra Katwal, Prakash Kayastha, Suraj Shrestha, Pradeep R. Regmi

**Affiliations:** a Department of Radiology and Imaging, Tribhuvan University Teaching Hospital; b Maharajgunj Medical Campus, Institute of Medicine, Kathmandu; c Department of Radiology and Imaging, Dadeldhura Subregional Hospital, Nepal

**Keywords:** ectopic ureter, endometrioma, herlyn-werner-wunderlich syndrome, OHVIRA, vaginal septum

## Abstract

Obstructed hemivagina and ipsilateral renal agenesis (OHVIRA) syndrome also known as the Herlyn-Werner-Wunderlich Syndrome is a rare embryological disorder associated with Mullerian and mesonephric duct abnormality.

**Case presentation:**

The cases presented describe the imaging (ultrasound and MRI) findings of four young females who presented with dysmenorrhea and urinary complaints. All of them had solitary kidneys with a didelphic uterus and unilateral hematometrocolpos. A proximally blind-ending ureter with distal ectopic insertion, transverse vaginal septum, and left-sided endometrioma was seen.

**Clinical discussion:**

OHVIRA syndrome is associated with duplicated uterovaginal structure with OHVIRA. Ultrasound is the first line of investigation; however, MRI better delineates the anatomy and assists in preoperative planning.

**Conclusion:**

This report highlights that earlier clinical suspicion and imaging diagnosis of OHVIRA is crucial to prevent adverse outcomes and treating complications.

## Introduction

HIGHLIGHTS

- Obstructed hemivagina and ipsilateral renal agenesis syndrome is a rare embryological disorder of the mesonephric and paramesonephric duct.
- MRI helps to better delineate anatomy and preoperative planning.
- An imaging diagnosis of obstructed hemivagina and ipsilateral renal agenesis can help prevent adverse outcomes.

The mesonephric duct and paramesonephric ducts are embryological structures that develop into the internal genital organ and urinary tract in both sexes^1–3^. The mesonephric duct is located medially and acts as an inducer to the paramesonephric duct. Both paramesonephric ducts grow medially crossing the mesonephric duct and fuse medially, developing into the fallopian tube, uterus, and upper two-thirds of the vagina^4^. Various type of paramesonephric duct anomaly related to the maldevelopment and non-fusion includes agenesis, hypoplasia, unicornuate, didelphys, bicornuate, septate, and arcuate uterus^5^. The lower one-third of the vagina develops from the urogenital sinus and later fuses with the Mullerian duct. The defect in fusion forms the vaginal septum. The majority of patients with a complete vaginal septum are associated with the uterus didelphys and a few with the bicornuate bicollis uterus^6^. The urinary and reproductive system abnormality occurs concurrently as they are closely associated embryologically. The ureteric bud that evolves from the mesonephric duct develops into the renal pelvis and renal collecting systems, while the mesonephric duct regresses in females leaving the epi-oophoron, para-oophoron, and Gartner’s duct cyst as the remnant^1–3^.

Obstructed hemivagina and ipsilateral renal agenesis (OHVIRA) syndrome also known as the Herlyn-Werner-Wunderlich Syndrome is a rare embryological disorder associated with Mullerian and mesonephric duct^7^. It is associated with duplicated uterovaginal structure with OHVIRA^8,9^. Renal agenesis with ipsilateral blind hemivagina was reported as Herlyn Meyer syndrome in 1971, while renal aplasia and bicornuate uterus with isolated hematocervix and simple vagina were reported by Wunderlich in 1976^10,11^. Patients with OHVIRA generally present with the symptoms of abdominal pain, pelvic mass, and dysmenorrhea as well as urinary urgency, frequency, and vaginal discharge^12,13^. Hematocolpos due to endometriosis may lead to pelvic pain, which can be treated by therapeutic intervention if diagnosed early. Early intervention also helps to maintain reproductive potential^11^. Although USG is the initial modality of choice for Mullerian duct anomalies due to its low cost, no radiation risk, and easy availability; however, relies mainly on the operator’s expertise^14^. Due to multiplanar capabilities, higher soft tissue resolution, and larger field, MRI can better study the uterine and vaginal contours, their content as well as any associated renal anomalies thus helping with the surgical planning^15^.

Herein, we present the imaging findings of four young females with a diagnosis of OHVIRA syndrome who presented with varying signs and symptoms. Axial, coronal, and sagittal MRI images were evaluated. This case has been reported in line with Surgical CAse REport (SCARE) criteria^16^.

## Case presentations

### Case 1

**Figure 1:**
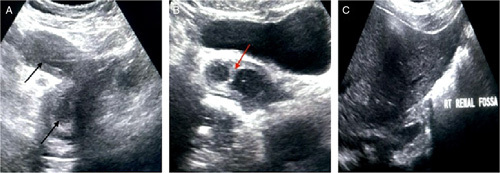
USG image of a 23-year-old female showing didelphic uterus (black arrow in 1(A), vaginal septum (red arrow in 1B), and absent kidney in right renal fossa (1C).

**Figure 2:**
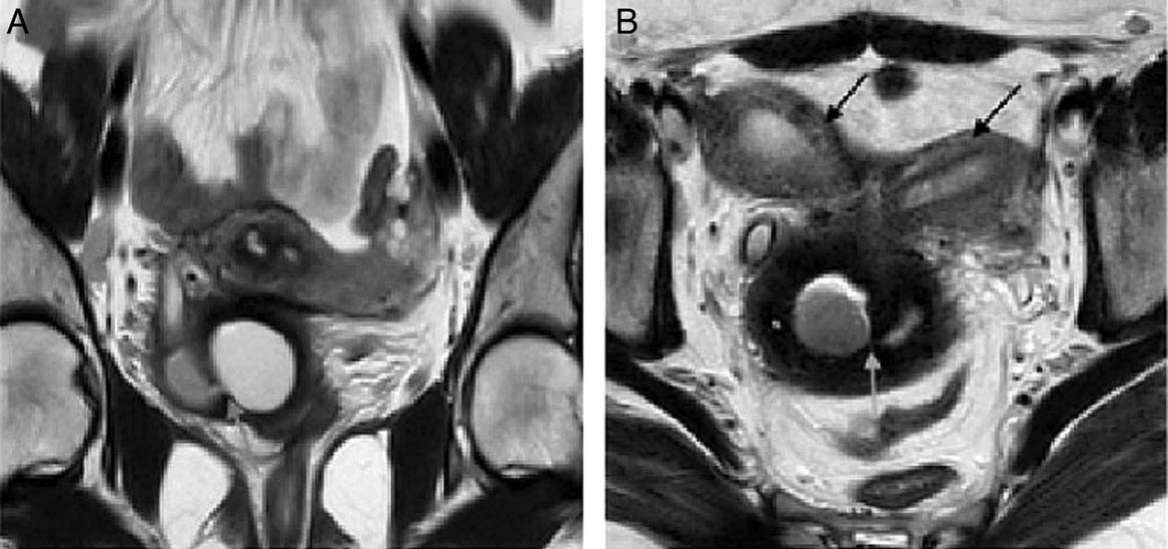
MRI T2-weighted coronal and axial image of a 23-year-old female showing a didelphic uterus (black arrow in 2B) and tiny communication between two hemivagina (white arrow in 2A and 2B).

A 23-year-old female, who had her menarche at the age of 14 years, presented with a history of recurrent lower abdominal pain predominately during the menstrual period. She underwent ultrasound imaging, which showed the didelphic uterus distended with an echogenic content (Fig. 1A, B) and the absence of the right kidney (Fig. 1C). MRI showed the uterine didelphys with obstructed right hemivagina and hematometrocolpos (Fig. 2A, B). The right kidney was not visualized. A tubular structure with a proximal blind-ending was noted with communication to the right hemivagina. The left vagina was patent and a small communication to the right hemivagina was noted. Drainage of the obstructed vagina creating a small window was done twice before. She is planned for septal resection later on in life.

### Case 2

**Figure 3:**
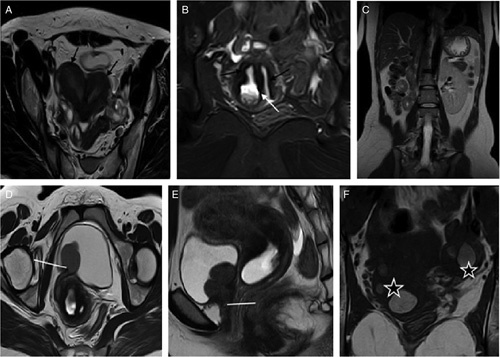
A 28-year-old female with irregular vaginal bleeding. Black arrows in 3A and 3B show the didelphic uterus and bilateral hemivagina, respectively, with a white arrow in Figure 3B showing the tiny communication. Agenesis of the right kidney is shown in Figure 3C. Dilated distal ureter with an ectopic opening in the urethra is shown by the white line in 3D and 3E. Bilateral endometrioma is shown by white stars in Figure 3F.

A 28-year-old female presented to the gynecology outpatient department with a complaint of irregular vaginal bleeding. She had her menarche at the age of 15 years. With the onset of menstruation, she started complaining of dysmenorrhea. Repeated episodes of urinary tract infection is also noted. She underwent ultrasound imaging which showed the didelphic uterus distended with echogenic contents along with an absence of the right kidney. MRI supported ultrasound findings, which revealed uterine didelphys with hematometrocolpos on the right side (Fig. 3A, B). A dilated right ureter with a blind-ending proximal end and distal ectopic opening of the right ureter into the urethra was noted (Fig. 3D, E). In addition, horizontal vaginal septa were noted on the right side with an obstructed right hemivagina, distended with blood signal, and tiny communication to the left hemivagina (Fig. 3B). Moreover, an ~2.8×1.7×1.5 cm-sized cystic lesion with a blood signal was noted in the left ovary (Fig. 3F) with a similar lesion in the right ovary along with agenesis of the right kidney (Fig. 3C).

### Case 3

**Figure 4:**
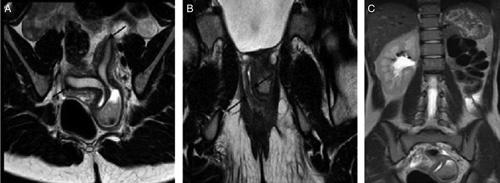
A 14-year-old female with dysmenorrhea. MRI shows (A) a Didelphic uterus (black arrow) with a distended left cervix by heterogenous signal content (B) Bilateral hemivagina with a collapsed lumen (black arrow). (C) Absence of left kidney.

A 14-year-old female presented to gynecological outpatient department with persistent dysmenorrhea despite taking over the counter drug. She had her menarche 2 years back. She also complained of urinary retention during the menses. An ultrasound examination showed a large cystic lesion in the pelvis with uterine didelphys and an absent left kidney. A subsequent MRI scan showed uterine didelphys with distended left hemivagina by blood signal content (Fig. 4A, B). The right hemivagina was patent with tiny communication to the left hemivagina and left renal agenesis was noted (Fig. 4C).

### Case 4

**Figure 5:**
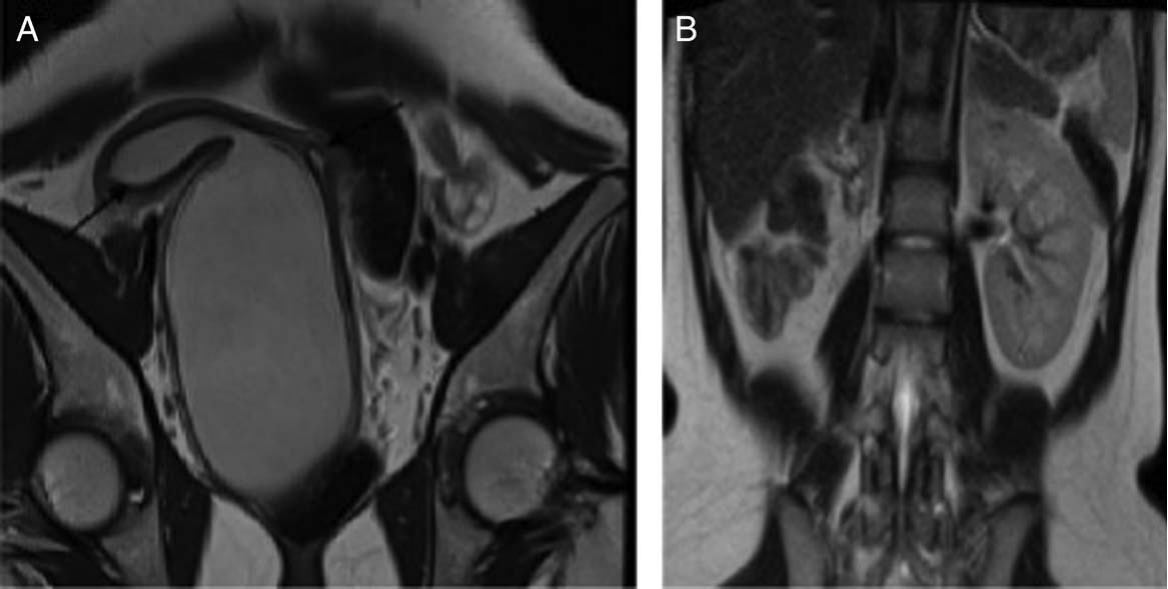
A 14-year-old female with dysmenorrhea. (A) Coronal T2-weighted image shows a didelphic uterus (black arrows) with high signal content in the right cervix and vagina. Mild distension of the right uterine cavity, left uterine, and cervical canal is collapsed. (B) Agenesis of the right kidney is shown.

A 14-year-old female presented with complaints of dysmenorrhea, incomplete voiding, and increased frequency of micturition. She had no menstruation for the past 4 months and had only light bleeding and vaginal spotting during the menstrual period. However, she had severe lower abdominal pain during menstruation. An ultrasound examination showed a large pelvic cystic lesion with the didelphic uterus. With suspicion of Mullerian duct anomaly, an MRI was advised which showed uterine didelphys with obliterated right hemivagina and right hematometrocolpos with right renal agenesis (Fig. 5A, B). The right hematometrocolpos was compressing and displacing the bladder anteriorly. A transverse vaginal band was seen about 2.5 cm above the introitus.

## Discussion

OHVIRA syndrome/Herlyn-Werner-Wunderlich Syndrome is a rare Mullerian anomaly with a prevalence of about 2–3%^17^. The most common renal anomaly associated with this disorder is agenesis, although dysplastic kidney or renal duplication may also be present^9^. All four cases of our study had renal agenesis, three on the right side and one on the left. OHVIRA syndrome is related to the impaired development of both mesonephric and paramesonephric ducts with renal agenesis related to the mesonephric and didelphic uterus with vaginal septum related to paramesonephric pathology^8^.

Clinically, the patient may present with abdominal pain, pelvic mass, dysmenorrhea as well as urinary symptoms like urgency, frequency, and vaginal discharge^13^. All our patients complained of dysmenorrhea. Among them, three had urinary symptoms like retention of urine, increased frequency, urgency, and burning micturition. Irregular vaginal bleeding with episodes of spotting was also noted. In addition, all our patients presented at an earlier age. It is difficult to achieve an accurate diagnosis because menstruation is often regular and when the patient complains of symptoms of cyclic dysmenorrhea, they are usually given anti-inflammatory drugs and oral contraceptives, thus causing a delay in the diagnosis because they reduce or eliminate menses, and thus OHVIRA is not often thought of as a diagnostic possibility^18^. In addition, various other causes of outflow tract obstruction, which include imperforate hymen, transverse vaginal septum, longitudinal vaginal septum, and cervical atresia pose additional diagnostic challenges^19^.

OHVIRA syndrome with an atypical presentation of cervical incompetence in pregnancy or ruptured uterus has also been reported^20^. Early diagnosis of this disorder is crucial to relieve the symptoms and prevent the worse outcome concerning the pregnancy like miscarriage, preterm birth, malpresentation, low birth weight, and caesarian delivery^21^. Also, there is a need for preconception counseling and good antenatal care to avoid perinatal morbidity and mortality. The associated renal anomaly may aggravate hypertension in pregnancy and iatrogenic injury to the ureteric remnant or ectopically inserted segment is also high^22^. Two of our patients had the ureteric remnant with ectopic insertion to the vagina and urethra, respectively.

Ultrasound is the first line of investigation that helps to look for renal agenesis, bicornuate uterus, and collection in the uterine or vaginal cavity. Ultrasound is also an easily available and cost-effective mode of investigation. Preoperative MRI is a better investigation modality that delineates the anatomical contour, uterine and vaginal morphology, location of the septum as well as urogenital assessment. Findings are helpful for surgical planning. MRI findings were found to be more consistent with intraoperative findings^23^. If findings in an MRI are inconclusive, laparoscopy is the gold standard modality. Because of an improved understanding of the disease and its association, minimally invasive vaginoscopic surgery-guided resection of the vaginal septum and drainage of the fluid is the mainstay of treatment nowadays^24^. All our cases showed the hematometrocolpos with the transverse vaginal symptom. The collection was compressing the bladder causing incomplete evacuation in one patient. There was tiny communication between the two vaginal septa, which helped for slow drainage of the collection. One of our cases had already gone windowing of the septum for drainage, which gave her symptomatic relief. She is being planned for the surgical resection of the septum while the rest of the patients were lost to follow-up.

## Conclusion

If an adolescent girl presented with dysmenorrhea or urinary symptoms, internal genital organs, and the renal system should be looked for. If renal agenesis is found, screening for Mullerian anomalies, and vice-versa, that is, when the spectrum of Mullerian agenesis is suspected, screening for renal anomalies should be done. This is imperative for the early diagnosis of OHVIRA, initiating the treatment, and preventing its adverse outcomes.

## Ethical approval

Not required.

## Consent for publication

Written informed consents were obtained from the patients for publication of this case report and accompanying images. A copy of the written consent is available for review by the Editor-in-Chief of this journal on request.

## Source of funding

None.

## Author contribution

S.P.: conceptualization, data curation, writing - original draft, writing - review and editing, supervision, project administration; S.K.: conceptualization, data curation, writing - original draft, writing - review and editing, supervision, project administration; P.R.R.: conceptualization, data curation, writing - original draft, writing - review and editing, supervision, project administration; P.K.: conceptualization, data curation, writing - original draft, writing - review and editing, supervision, project administration; S.S.: writing - original draft, writing - review and editing.

## Conflict of interest

None to declare.

## Research registration unique identifying number (UIN)

Not Applicable.

## Guarantor

Dr Suraj Shrestha.

## Data availability statement

All the necessary information are provided within the manuscript.

## Provenance and peer review

Not commissioned, externally peer-reviewed.
